# Self-management at the core of back pain care: 10 key points for clinicians

**DOI:** 10.1016/j.bjpt.2021.05.002

**Published:** 2021-05-24

**Authors:** Alice Kongsted, Inge Ris, Per Kjaer, Jan Hartvigsen

**Affiliations:** a Department of Sports Science and Clinical Biomechanics**,** University of Southern Denmark**,** Odense M**,** Denmark; b Chiropractic Knowledge Hub**,** Odense M**,** Denmark; c Health Sciences Research Center**,** UCL University College**,** Odense M**,** Denmark

**Keywords:** Back pain, Behavior change, Delivery of health care, Musculoskeletal disease, Patient-centred Care, Self-management

## Abstract

**Background:**

A paradigm shift away from clinician-led management of people with chronic disorders to people playing a key role in their own care has been advocated. At the same time, good health is recognised as the ability to adapt to changing life circumstances and to self-manage. Under this paradigm, successful management of persistent back pain is not mainly about clinicians diagnosing and curing patients, but rather about a partnership where clinicians help individuals live good lives despite back pain.

**Objective:**

In this paper, we discuss why there is a need for clinicians to engage in supporting self-management for people with persistent back pain and which actions clinicians can take to integrate self-management support in their care for people with back pain.

**Discussion:**

People with low back pain (LBP) self-manage their pain most of the time. Therefore, clinicians and health systems should empower them to do it well and provide knowledge and skills to make good decisions related to LBP and general health. Self-management does not mean that people are alone and without health care, rather it empowers people to know when to consult for diagnostic assessment, symptom relief, or advice. A shift in health care paradigm and clinicians’ roles is not only challenging for individual clinicians, it requires organisational support in clinical settings and health systems. Currently, there is no clear evidence showing how exactly LBP self-management is most effectively supported in clinical practice, but core elements have been identified that involve working with cognitions related to pain, behaviour change, and patient autonomy.

## Highlights

• Self-management support is person-centred care reinforcing patient autonomy.
• Avoid strong clinician control and help patients developing self-efficacy.
• Let patients’ value-based goals and shared decisions guide management.
• Help patients make sense of symptoms and reframe unhelpful perspectives.
  Use supervised exercises as a tool to practice problem-solving skills

## Background

Across non-communicable chronic conditions, a paradigm shift away from clinician-led management towards management where people with chronic conditions play a key role in their own care is advocated.^1^^,^^2^ At the same time, good health is increasingly understood as the ability to adapt to changing life circumstances and to self-manage in the face of social, physical, and emotional challenges.^3^^,^^4^ In the case of persistent low back pain (LBP), such an approach implies that successful interventions are not mainly about clinicians diagnosing and curing patients, but about a partnership between individuals and clinicians that helps people engaging in valued activities.^5^ Thus, living with persistent or recurrent LBP may involve care-seeking, but people manage their health conditions outside the context of health care most of the time, and interventions for persistent LBP should enable them to do that well.

In this paper, we discuss self-management in relation to LBP with a focus on the role of the clinician.

## Self-management terms

**Figure 1:**
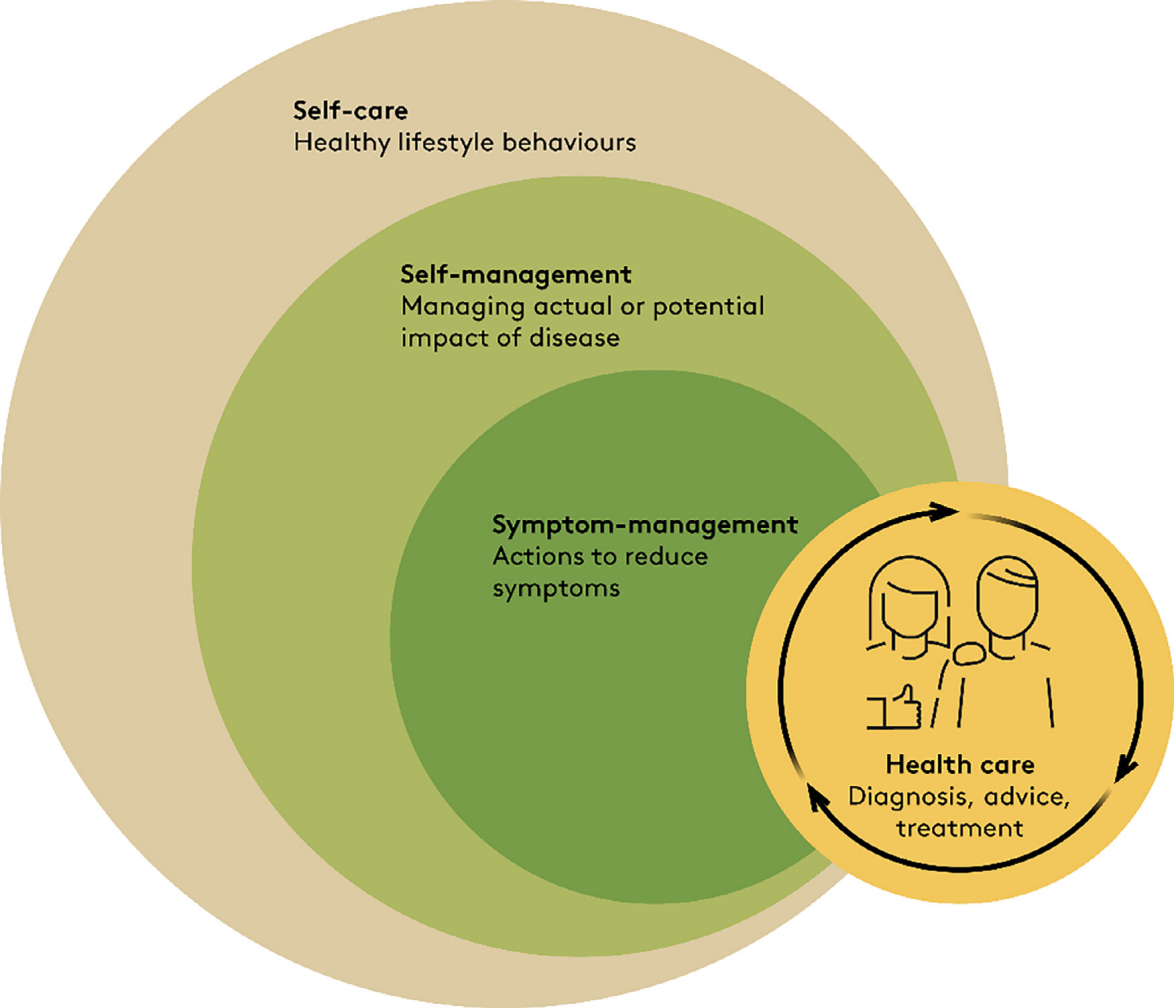
How self-care, self-management, symptom-management, and health care are related. Self-management of disease, including symptom-management, is part of self-care and may be performed in collaboration with health care providers. Illustration based on Richard and Shea.^10^

Among clinicians and within the literature, terms like self-care, self-management, self-efficacy, and symptom management are often used interchangeably without clear definitions and without presentation of the underlying theory.^6^^,^^7^ Therefore, we briefly introduce how we use these terms (Fig. 1).

*Self-care* is all the actions that people do to stay healthy (e.g. brushing teeth, sleeping well, eating healthy food), and includes actions that aim to prevent disease, maintain good health, and coping with illness and disability.^8^ *Self-management* has been defined as “the individual's ability to manage the symptoms, treatment, physical and psychological consequences, and lifestyle changes inherent in living with a chronic condition,” and is the part of self-care that relates to dealing with health conditions.^9^, ^10^, ^11^ Definitions of self-management emphasize the importance of interactive, collaborative care between patient and healthcare professionals allowing for patient empowerment rather than one-way passive care from expert to patient.^10^, ^11^, ^12^*Symptom management* is the actions initiated by the patient, a clinician, or both to decrease the distress and consequences caused by symptoms. It entails a collaborative relationship between a patient and the healthcare provider to make decisions about for example medication or manual therapy interventions.^9^

Self-care and self-management are concepts with ties to Orem's theory and Bandura's Social Cognitive Theory on self-efficacy.^13^, ^14^, ^15^ *Self-efficacy* is people's beliefs in their ability to influence events that affect their lives. This core belief is the foundation of human motivation, performance accomplishments, and emotional wellbeing. Unless people believe they can produce desired outcomes by their actions, they have little incentive to undertake activities in the face of difficulties. Whatever factors may serve as guides and motivators, they are rooted in the core belief that one can make a difference by one's actions.

The focus of this paper is on self-management of persistent LBP where this involves the interaction and communication between the healthcare provider and the patient in a clinical encounter, and we describe the engagement of the clinician as ‘self-management support’.^9^ Self-management support is provided in self-management interventions defined as interventions that “aim to equip patients with skills to actively participate and take responsibility in the management of their chronic condition to function optimally.”^12^

## Why should clinicians care about self-management in LBP?

Most people who experience LBP will have recurrent episodes with pain that comes and goes.^16^ Even patients who recover well from an episode of LBP most likely will experience new episodes, and up to 20% of those seeking care for LBP have persistent LBP that they need to manage more or less continuously.^16^

The impact of LBP on daily activities differs substantially between individuals for reasons that are not fully understood. However, people are more disabled from LBP when they perceive their condition as frightening and out of their control and have low pain self-efficacy.^17^ Also, effective interventions for persistent pain conditions work partly by influencing beliefs, catastrophising thoughts, fear, and pain self-efficacy.^18^, ^19^, ^20^, ^21^ This includes interventions that are designed to have physical effects,^22^^,^^23^ implying that cognitions and emotions are not only affected by psychological interventions.

A traditional biomedical paradigm would focus on structural and degenerative changes in the spine that presumably explain the patient's symptoms. However, these do not correlate well with an individuals’ pain or activity limitations,^24^^,^^25^ although associated with an increased risk of LBP in populations.^26^ They also do not inform what treatment the patient most likely will respond to, nor do they inform the prognosis.^27^^,^^28^ A structural diagnosis does therefore not help patients make sense of their symptoms, and it may, in fact, add to their fear and worry, and even drive the use of ineffective treatments.^29^, ^30^, ^31^, ^32^, ^33^ Although exercises are often prescribed to improve muscle function and mobility there is little evidence that those are the mechanisms behind positive clinical effects.^34^, ^35^, ^36^

*Clinicians should care about self-management because most people with LBP continuously manage their condition and should be enabled to do it well. Supporting self-efficacy is an important element of LBP care because people are less disabled by LBP if they trust in their ability to manage it, and effective treatments for LBP partly work by reducing fear and increasing self-efficacy.*

## How can clinicians integrate self-management support in LBP management?

**Figure 2:**
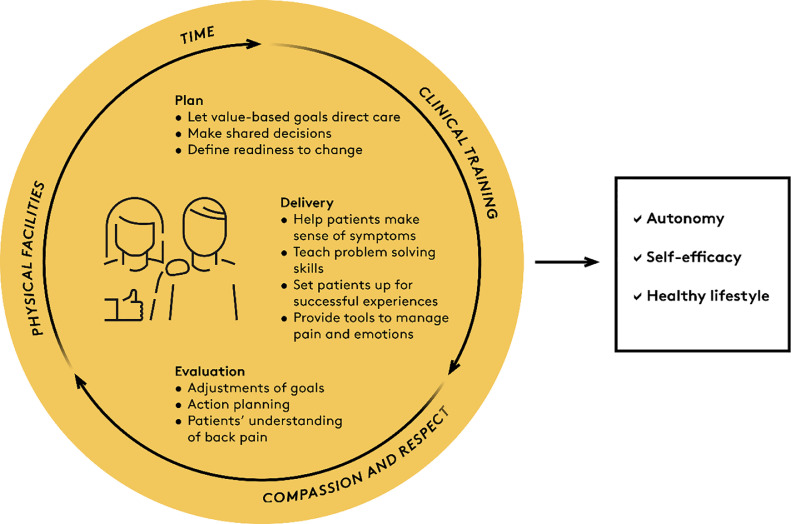
Clinical actions in self-management support. Self-management support includes actions related to intervention planning, delivery, and evaluation, and places demands on clinicians and organisations.

Clinical guidelines generally recommend advice and information, manual therapy, and supervised exercises as treatments for persistent LBP.^37^, ^38^, ^39^ These interventions are effective parts of symptom management and may prevent relapse, but do not necessarily support patient autonomy and self-management. Below, we outline clinical actions that help integrate self-management support in LBP management including behaviour change techniques (i.e. strategies to help patients adopt healthy behaviours) that are frequently incorporated in self-management interventions, and actions to focus on patient autonomy and self-efficacy (Table 1).^12^^,^^40^, ^41^, ^42^ These actions are aligned with intervention planning, intervention delivery, and clinical evaluation (Fig. 2).

**Table 1:** Actions to place self-management at the core of back pain care.

Clinical process	Self-management component	How?	Why?
Planning	Let patient value-based goals guide management.	Discuss specific patient value-based goals using for example the SMART framework.	To help patients identify their motivation for change and what facilitates and hinders engaging in valued activities. To reduce focus on pain-goals.
	Make shared decisions about the plan.	Exchange information about treatment options. Include patients’ values and preferences. Affirm the decision.	To enhance patient-centred care and increase patients’ satisfaction, engagement, and adherence.
	Define readiness to change.	Identify resources and knowledge to make a lasting change successfully. Identify barriers to change. Identify challenges to maintain new behaviour.	To understand elements of change, the stages of change, and ways to address each stage to achieve goals.
Delivery	Help patients make sense of their symptoms.	Deliver knowledge to reduce fear and address misconceptions. Direct patients to useful sources of information.	To avoid restrictions in patient-valued activities and low sense of control due to misbeliefs and fears.
	Teach skills to solve everyday problems.	Use exercises as a tool to train problem-solving skills by patients exploring movement instead of being told what to do. Encourage patients to try out a variety of movements and activities. Help patients come up with solutions to everyday problems.	To avoid dependency on the clinician, as being the one knowing what correct movement/posture is. To enable patients to cope with everyday situations on their own To increase self-efficacy by using operant conditioning, positive reinforcement and positive experiences.
	Set patients up for successful experiences	Use exposure to new/feared activities to provide the experience of success. Discuss alternative perspectives on feared activities or movements.	To help patients reframe their perspectives on low back pain. To challenge overly negative beliefs. To change thoughts or emotions related to an activity.
	Provide tools for management of pain and emotions	Teach pain strategies as distraction, breathing exercises, or mindfulness.	To support planning of active behaviour with relapses and flare ups. To enhance feeling of control.
Evaluation	Evaluate and discuss adjustments of goals	Ask if the patient-valued goals have been achieved: Partly: “What went well?” Not at all: “What are the barriers?”	To keep focusing on value-based goals and motivate for maintained engagement.
	Evaluate patients’ understanding of back pain	Ask questions about patients’ beliefs related to pain and what forms those beliefs: “What do you think happens when your back hurts?”	To raise awareness of more appropriate back pain beliefs.
	Assist patients in action planning	Discuss active tools to maintain self-managing of back problems. Prompt detailed planning of actions to take when perusing goals is challenged “How will you react when you back relapses/has flare ups?” “When will you need help from health care and why?”	To help patients sustain good habits and prepare for relapses.

## Self-management: planning

### Let patients’ value-based goals direct care

Individual goal setting, such as the ‘SMART’ (Specific Measurable Achievable Realistic Time-bound) method, helps patients identify their motivation for change, increase adherence to their plan, and helps clinicians plan interventions that support these goals.^43^ Value-based goal setting can open the communication about people's motivation for change and can reveal what facilitates and hinders reaching these goals.^44^ For example, it could be an underlying premise for a patient that the pain needs to be reduced for the patient to engage in valued activities. Through dialogue and reflections, the patient may, however, realise that pain beliefs or emotions are more central barriers for activity than the pain itself. Therefore, goals that relate directly to pain such as “I want to get rid of my back pain”, may lead to stress and frustration instead of action and obstruct patients from pursuing other and more valued goals.^45^

### Make shared decisions

Intervention planning and goal setting should optimally be based upon a shared decision-making process between patient and clinician. This process aims at balancing the patients’ right to autonomy with the clinicians’ responsibility to protect patients’ safety and ensure best-evidence care. For a shared-decision process to take place, the first requirement is to make it explicit that a decision has to be made.^46^ Thus, patients should know that there are different options and should be provided with best-evidence information that will help them make an informed decision on what their preference is. Shared decision making is part of patient-centred care and a way to increase engagement, patient satisfaction, and adherence.^47^ Shared decision-making is, however, challenging to implement in practice and requires that clinicians are well-informed about a patients’ options for care and have strong communication skills.^48^

### Define readiness to change

Because change does not happen at once and has to be driven by patient engagement, the patient's readiness to change their behaviour needs consideration to define achievable and realistic goals and action plans. For this purpose, five stages of behavioural change have been proposed: Precontemplation (unawareness or denial with no intention of changing behaviours), Contemplation (ambivalent about possibilities to change), Preparation (action planning, start changing behaviour), Action (changing behaviours, using self-management strategies but not adopted as a new habit), and Maintenance (consolidating new behaviour and self-management strategies in everyday life).^47^^,^^48^ Patients in the first 2 or 3 stages may need more information and education, whereas those in the last stages may need reassurance and positive feedback.

*Self-management support can be integrated into intervention planning by letting patient value-based goals and a focus on behaviour change direct management while shifting focus away from structure, pain, and impairments.*

## Self-management: delivery

### Help patients make sense of their symptoms

Although empirical evidence is sparse, changing LBP related behaviours seems intimately related to changing beliefs.^49^, ^50^, ^51^ Patients perceive LBP as unpredictable and uncontrollable and difficult to make sense of, which hampers their ability to deal with it in an expedient way.^31^ Educating patients about pain mechanisms and management may therefore prevent them from restricting their valued activities because of misbeliefs and fears. There are many useful pain education resources directed at clinicians and people with pain (see references for suggested readings, videos, podcasts and web sites^52^, ^53^, ^54^, ^55^, ^56^, ^57^, ^58^, ^59^, ^60^, ^61^, ^62^, ^63^), however, there is also a lot of misinformation about LBP.^64^ Therefore, clinicians should direct patients to suitable sources of information where inappropriate messages and pain education using terminology relating to spinal instability, postural abnormalities, wear and tear, discs “popping” in and out, or restrictions on what patients are ‘allowed’ to do or not are avoided.

### Teach problem-solving skills

Supervised exercises can be used as a tool to practice problem-solving skills.^65^^,^^66^ When patients experience pain during an exercise, difficulties in performing desired movements, or fear about their consequences, the clinician has an opportunity to explore their thoughts about causes and consequences by encouraging patients to experiment with moving in different ways. Exercises then become behavioural experiments that help patients reframe their beliefs and emotions related to activity. Traditionally, however, clinicians often correct patients when they perform exercises, based on the assumption that benefits of exercises depend on performing movements in very specific ways, when in fact there is little evidence to support that the way exercises are performed relates to outcomes. Importantly, correcting patients carry a risk of decreasing self-management skills by communicating that exercising is difficult and potentially unsafe to do on your own. Thus patients may lose autonomy and self-efficacy and become fearful of doing something “wrong” or potentially harm themselves.

### Set patients up for successful experiences

Re-engaging in valued activities may involve exposure to movements and activities that have been avoided. Here, graded exposure ensuring that progression feels safe or to gradually increase physical performance can be helpful. If exposure is a tool to reframe beliefs about consequences, it should include exposures to tasks, postures, or movements that have been avoided.^51^ This exposure is an opportunity to provide a positive experience and increase the patient's beliefs in their ability to move and be active. Operant conditioning principles, stating that pain behaviour is reinforced if these behaviours result in pain reduction or positive attention from others, can also be used to reinforce healthy behaviour by increasing activity gradually in a time-contingent manner.^67^, ^68^, ^69^ Using operant conditioning, activity, or exercises should not be directed by pain as this would reinforce withdrawal from activity.

### Provide tools to manage pain and emotions

Living with LBP invariably involves episodes of flare-ups and situations with increased pain. Therefore, patients need a ‘toolbox’ for managing pain and related fears or other emotions which includes tools such as distraction and breathing exercises,^70^ mindfulness techniques,^71^^,^^72^ or walking.^72^

*Self-management support is integrated into the delivery of the intervention when clinicians help patients making sense of their symptoms, discuss pain behaviours, and avoid supporting negative beliefs. Clinicians can use active interventions to teach problem-solving skills and provide patients with insights and tools to better manage their pain and overcome obstacles encountered in everyday life.*

## Self-management: evaluation

### Evaluate goals and patients’ understanding of back pain

Re-assessment and reflection are necessary to evaluate treatment outcomes and for clinicians’ ongoing learning process, and therefore an integrated part of health care. Evaluation of patients’ progress must be aligned with the intention of care, so the evaluation of self-management interventions should include assessment of patients’ understanding of their symptoms as well as achievements of patients’ individual goals and discussion about strategies and needs for adjusting these.

### Assist patients in action planning

The patient should be encouraged to make an action plan for dealing with future challenges and relapses. Here, patients’ stage of change of behaviour should be evaluated and the action planning related to this.

*Clinicians support self-management by evaluating patient valued goals, action plans, and phase of change instead of defining success as a cure of symptoms.*

### Pre-requisites

Integrating self-management support into routine care requires organisational support (Fig. 2). First, clinicians need training in communication skills, behavioural change techniques, and in working with patient-centred care as this is often not a part of their basic training.^73^ Then, there is a need for a practical clinical set-up that allows for self-management support including having sufficient time for dialogue and facilities that protect patient confidentiality when discussing personal matters. Finally, health systems need to support clinicians by providing reimbursement for time spent on patient education and on promoting behaviour change.^74^

Notably, shifting the paradigm of care requires that clinicians are open to thinking differently about LBP care throughout the clinical encounter. Table 2 lists some “*dos and don'ts*” illustrating that translating treatment into self-management support may require profound shifts in clinical cognition and habits.

**Table 2:** Turning clinical activities and treatment into self-management support.

		Do…	Don't…
Planning	Defining success	Support and guide patients towards their individual value-based goals.	Communicate that cure of symptoms is the ultimate goal of treatment.
	Clinical assessment	Assess (pain) behaviours, impairments, thoughts, and feelings that facilitate and hinder valued goals.	Emphasize a structural diagnosis that does not inform choice of treatment and prognosis, or understanding of pain.
	Intervention plan	Provide evidence-based knowledge to help patients make informed decisions about their care.	Decide what is best for a patient or make them believe that their pain will be cured and never return.
Delivery	Patient education	Focus on the benign aspect of low back pain.	Focus on structural injury as an explanation of non-specific low back pain.
	Manual therapy	Include manual therapy in symptom management if valued by patients and helpful for achieving patient-valued goals.	Tell patients that their back pain cannot improve without manual therapy, that something is out of place or that manual treatment corrects spinal abnormalities.
	Exercise supervision	Use exercises to help people become confident with natural and varied movements and use exercises to develop problem-solving skills.	Tell patients that their back pain cannot improve without specific exercises, and don't make movement difficult by correcting them to achieve a ‘neutral posture’, ‘alignment’, or other clinician determined criteria for moving correctly.
Evaluation	Define success of intervention	Use patient-centred goals to define results of the intervention.	Use pain measurements to define results of the intervention.

*Self-management support requires organisational change and support from payers, educators, and clinic owners.*

## What is the evidence for self-management interventions in people with LBP?

Systematic reviews summarizing the evidence for the effectiveness of self-management interventions in people with persistent LBP report that there is considerable heterogeneity between studies and that the methodological quality is generally low to moderate. Nonetheless, across randomized clinical trials, interventions to promote self-management are generally found to have small to moderate effects on key clinical outcomes such as pain intensity, back-related disability, and self-efficacy at least up to one year post-intervention.^40^^,^^75^, ^76^, ^77^

Du et al.^40^ identified core elements of self-management interventions across trials that included problem-solving skills, decision making, resource utilization, a focus on the patient-clinician relationship, goal setting, and activity planning. They further found that generally, self-management interventions based on a theory, for example, the fear-avoidance theory, or delivered according to a theory, for example, cognitive behavioural therapy or social cognitive theory, were more effective than interventions that were not based on or delivered according to a theoretical framework; that interventions of shorter duration (<6 weeks^40^ or ≤8 weeks^78^) tended to be more effective than longer-lasting interventions; and that interventions where the whole or parts of the intervention was delivered over the internet or other eHealth platform were as effective as interventions delivered in person.^40^ Interventions delivered over mobile devices seemed superior to interventions delivered over the internet via web-pages, but these types of delivery had not been directly compared. There were also no trials directly comparing eHealth delivery to person delivery of identical self-management interventions.^78^ There is a lack of evidence to tell if self-management is best supported by individual or group-based interventions. A systematic review found group-based interventions for LBP to be more effective than other types of care for pain relief,^79^ whereas individual cognitive functional therapy for people with chronic LBP was more effective in reducing disability than group-based exercise and education in a recent trial.^80^

Despite a growing body of literature, most interventions aiming to promote self-management in people with LBP are not well described in reports. These interventions are often multifaceted and complex, so standardised reporting, for example according to the Criteria for Reporting the Development and Evaluation of Complex Interventions in healthcare (CReDECI), should be adopted.^81^ In addition, authors generally report on clinical outcomes such as pain intensity and back-related disability, but less often on behaviour related outcomes such as self-management skills, learning, and knowledge, which are more related to the goals of these interventions.^40^^,^^75^^,^^82^ One reason for this is the lack of valid measurements tool to capture the complexity of being able to self-manage.

*Current evidence on self-management interventions for LBP are hampered by a lack of theoretical frameworks and interventions are often poorly described. Still, the existing evidence suggests positive effects on a range of key outcomes and that eHealth may have an important role to play.*

## LBP care does not work in isolation

Health care is just one component in a person's strategies to manage their health, and people with persistent LBP very often have multiple chronic conditions.^83^ Therefore, self-management skills beyond coping with LBP are often needed to maintain a healthy life.

Results from 53 qualitative studies investigating patients’ perspectives on self-management of chronic diseases demonstrated that personal factors (e.g. knowledge, beliefs, and motivation), health status (including symptom severity and general health), available resources, social context (work, family, community), and health care systems all affect a person's ability to self-manage.^84^ Another synthesis of qualitative studies demonstrated that it can be an exhausting effort to maintain ongoing self-management of LBP, and the success of this sustained process depends on individual personal factors as well as support from clinicians, family, and friends.^85^ Thus health care does not act in isolation, and self-management strategies must be based on individual needs and care must be person-centred. Four principles of person-centred care have been described by The UK Health Foundation as: (1) affording people dignity, compassion, and respect; (2) offering coordinated care; (3) offering personalised care, and; (4) supporting people to recognise and develop their strengths and abilities to enable them to live an independent and fulfilling life.^86^

*Self-management interventions should support people's self-efficacy and autonomy not only as a tool for managing pain but rather support the ability to maintain good overall physical and mental health. People manage very different life situations and individual resources and contexts must be met with compassion and respect.*

## Summary

People with LBP self-manage their pain most of the time. Therefore, clinicians and health systems should empower them to do it well and provide knowledge and skills to make good decisions related to LBP and general health (Table 3). Self-management does not mean that people are alone and without health care, rather it empowers people to know when to consult for diagnostic assessment, symptom relief, or advice. A shift in health care paradigm and clinicians’ roles is not only challenging for individual clinicians, it requires organisational support in clinical settings and health systems. Currently, there is no clear evidence showing how exactly LBP self-management is most effectively supported in clinical practice, but core elements have been identified that involve working with cognitions related to pain, behaviour change, and patient autonomy.

**Table 3:** Summary of key aspects of self-management support.

Principles
**People self-manage most of the time** Enable them to do it well
**Support patient autonomy** Act as a partner, be person-centred, avoid strong clinician control
**Help patients develop self-efficacy** Provide and reinforce positive experiences
**It is not only about back pain** Helping people to manage their pain in daily life supports their ability to maintain good physical and mental health
**Working with self-management requires the right setting** Trained clinicians, time for dialogue, and room for confidentiality are pre-requisites for self-management support

**Clinical actions**
**Let patient value-based goals guide management** Focus on patient valued goals and shared decision making rather than on pain and dysfunction
**Help patients make sense of their symptoms** Educate people about pain and pain behaviour and help them reframe negative perspectives
**Teach skills to solve everyday problems** Give patients insights to help them overcome obstacles in everyday life
**Provide tools for pain management** Focus on proactive pain behaviour techniques
**Evaluate patient valued goals, action plans, and patients’ understanding of back pain** Move focus away from defining success as curing pain forever

## Declaration of Competing Interest

The authors have no conflict of interest to declare. AK's position at University of Southern Denmark is partly funded by the Foundation for Chiropractic Research and Post-graduate Education. IRH's position is financially supported by income from clinician training in GLA:D Back.
